# Spatial distribution and characteristics of martial arts halls in China

**DOI:** 10.3389/fpubh.2025.1695782

**Published:** 2025-12-01

**Authors:** Yunfeng Yao, Shuangrui Liu, Pengfei Yu, Wanyi Wu

**Affiliations:** 1 College of Physical Education and Health, East China Normal University, Shanghai, China; 2 College of Physical Education, Kyungpook National University, Daegu, Republic of Korea; 3 Physical Education Department, Physical Education School, East China University of Technology, Nanchang, China; 4 College of Sports Humanities and Social Sciences, Wuhan Sports University, Wuhan, China

**Keywords:** martial arts hall, martial arts culture, martial arts industry, spatial distribution, China

## Abstract

**Introduction:**

The spatial distribution of martial arts halls plays a vital role in the inheritance and dissemination of Chinese Wushu culture. As important spaces for preserving martial arts traditions, these halls embody cultural continuity and serve as tangible carriers of intangible heritage. However, the nationwide spatial distribution characteristics and their underlying influencing factors remain insufficiently explored.

**Methods:**

Using spatial geography techniques and data from 5,805 martial arts halls across China as of March 2025, this study employs kernel density estimation, spatial autocorrelation analysis, and regression models to examine the spatial distribution patterns and determinants of martial arts halls.

**Results:**

The results reveal significant regional disparities, with high concentrations in eastern provinces and sparse distributions in the west. The spatial structure is characterized as a “Four Core–Ring–Core Group–Double Belt” pattern. Population size, martial arts cultural heritage, policy support, and education level exert positive influences on hall distribution, whereas competition from the cultural and recreational industries poses challenges. Economic factors demonstrate a dual effect, acting both as a driver and constraint.

**Discussion:**

The findings highlight that the spatial development of martial arts halls reflects broader socio-economic and cultural patterns in China. To enhance their sustainable development and promote balanced regional growth, policy interventions should strengthen cultural inheritance mechanisms, optimize resource allocation, and encourage integration between martial arts education and local development strategies.

## 1 Introduction

### 1.1 Culture and policy background

Martial arts (Wushu) are not merely a codified repertoire of techniques but a living form of intangible cultural heritage whose meanings are continually reproduced through practice, place, and pedagogy. As such, Martial arts participates in broader global debates on safeguarding embodied knowledge, where the vitality of tradition depends on sustaining both communities of practice and the spatial infrastructures that anchor them. International policy frameworks—most prominently UNESCO’s 2003 Convention for the Safeguarding of the Intangible Cultural Heritage—have elevated the preservation of such practices to a transnational priority (1–4). In step with these developments, China has advanced a multi-pronged agenda that couples cultural stewardship with sectoral development, providing support through policy, cultural programming, and market mechanisms so as to align national objectives with international heritage discourse (5–7). Within this policy milieu, the martial arts hall (MAH) has been substantively reconfigured. No longer a purely traditional training venue, the MAH now operates as a strategic node at the intersection of cultural heritage transmission, the sports industry, and market logics. China’s “Wushu Industry Development Plan (2019–2025)” explicitly calls for an integrated industrial ecosystem that links fitness, performance, training, and research, while accelerating the development of high-quality MAHs as core infrastructure (6). This institutional realignment has amplified the spatial stakes of MAHs: they function simultaneously as carriers of cultural reproduction, as anchors of local cultural economies, and as service hubs whose siting and accessibility condition who participates in, and benefits from, Martial arts.

Scholarly inquiry in China has laid important groundwork on the spatial dimensions of martial arts and related facilities. Existing studies document correlations between sports venues and macro-economic indicators, identify management challenges specific to MAHs, and map the geographic distribution of individual martial arts styles from folkloristic perspectives (7–9). Research has also delineated the spatial characteristics of designated “Wushu Hometowns” and school systems (10, 11). While valuable, this body of work has tended to privilege single cases, descriptive mapping, or managerial lenses, often at the expense of comprehensive, multi-scalar, and quantitatively rigorous assessments that integrate spatial form, locational scale, and distributional patterns with demographic and economic structures.

A comparative international perspective indicates that Sports Geography and Cultural-Heritage Geography provide mature conceptual and methodological resources directly relevant to MAHs. Foundational sport-place scholarship has theorized “sportscapes” and demonstrated that facility distributions are patterned—not random—by policy, demography, urban hierarchy, and identity-laden symbolic geographies. Empirical studies routinely model the socio-economic and demographic determinants of facility location and access across arenas ranging from elite stadia to neighborhood parks and fitness centers (12, 13). In parallel, heritage studies increasingly deploy Geographic Information Systems (GIS) to map, manage, and analyze spatial patterns of both tangible and intangible heritage, including the clustering of creative industries, the regional spillovers of heritage sites, and the spatial dynamics of cultural practice transmission (14, 15). Methodologically, international precedents have consolidated a toolkit—e.g., mean nearest neighbor indices, kernel density estimation, spatial autocorrelation, and spatial/regression frameworks—that is well suited to diagnosing agglomeration, diffusion, and service equity in heritage-sport hybrids such as MAHs.

Against this background, the present study applies spatial analytical techniques and regression modeling to interrogate the national-scale spatial ecology of martial arts culture (MAC) dissemination in China and to characterize the supply structure of associated education and training services. By explicitly linking facility distributions to population structure and economic development while attending to multi-level spatial organization, the study advances a more systematic, comparative perspective than prior descriptive or single-case approaches. The anticipated contributions are twofold: theoretically, to situate MAHs within an integrative framework bridging sports geography and heritage geography; and practically, to furnish evidence-based implications for martial arts -industry strategy and for the rational, sustainable spatial planning of the sector (6, 11).

## 2 Data and methods

### 2.1 Data processing

We use Python program to obtain the geographic location information of MAHs. The names and geographic coordinates of 6,146 MAHs were obtained in Baidu Map by using the keywords of “wushu hall,” “martial arts,” “martial arts hall” and “boxing hall.” To improve the accuracy of the sample, we used keywords such as “physical fitness,” “hip-hop,” “boxing,” “karate,” “taekwondo” and “sports training center” to screen out irrelevant institutions, and manually checked whether the addresses met the requirements in Baidu Map Street View mode^1^ one by one to form a vector database of contemporary martial arts halls as of Mar 2025. Finally, 5,805 valid samples were selected (Table 1). This study excludes the regions of the Hong Kong Special Administrative Region, the Macao Special Administrative Region, and Taiwan Province from its geographical scope. The economic and educational systems in these regions differ significantly from those in mainland China, making it inappropriate to analyze the factors influencing the spatial distribution of martial arts halls on the same basis. Therefore, these regions were not included in the analysis.

**Table 1:** Summary of the number of martial arts halls in each province.

Province	Quantity	Province	Quantity	Province	Quantity	Province	Quantity
Shandong	592	Anhui	199	Heilongjiang	102	Tianjin	58
Guangdong	586	Hubei	189	Inner Mongolia	89	Gansu	46
Henan	573	Fujian	185	Yunnan	87	Ningxia	35
Jiangsu	312	Sichuan	185	Beijing	85	Xinjiang	35
Hebei	306	Shaanxi	173	Jilin	85	Hainan	16
Hunan	253	Jiangxi	151	Chongqing	85	Qinghai	5
Zhejiang	251	Shanxi	134	Guizhou	83	Tibet	0
Liaoning	211	Guangxi	118	Shanghai	63	-------	---

### 2.2 Nearest neighbor index (NNI)

The nearest neighbor index can judge the aggregation or dispersion trend of spatial arrangement by comparing the actual average observation distance and the theoretical expected average interval between similar elements, which is suitable for evaluating the dispersion or dispersion trend of MAHs as a point element in geographical space. By comparing the actual average observed distance between MAHs with the theoretically expected average distance, this index can reveal the distribution pattern of MAHs in different regions of China. Considering that the geographical distribution of MAHs may reflect the popularity of MAC and regional cultural characteristics, this index is of great significance for understanding the geographical spread of MAC. In this study, ArcGIS 10.7 was used to calculate the nearest neighbor distance of the spatial entity of MAHs, and Z-Value test was used to describe the spatial distribution pattern of MAHs in China. The nearest neighbor index can be calculated by the formula (16):


$$R=\frac{R_{i}}{R_{e}}=\frac{1}{n}\sum_{i=1}^{n}d_{i}(s_{i})\times \frac{1}{2\sqrt{\frac{n}{A}}}$$

R is the nearest proximity index; $R_{i}$ represents the actual average nearest neighbor distance of MAH; $R_{e}$ is the theoretical nearest neighbor distance. $d_{i}(s_{i})$ represents the distance from the MAH to its nearest MAH; A is the geographical area of China; n is the number of MAHs. When R =1, it means that the distribution type of contemporary MAH is random model. When R >1, it was a uniform distribution type. When R <1, it is clustered distribution type.

### 2.3 Geographical concentration index (GCI)

The geographical concentration index is mainly used to assess the degree of concentrated distribution of point feature elements in a region. The geographical concentration index is used to evaluate the concentration distribution of MAHs in each region, and this index is especially suitable for revealing regional differences in the distribution of MAHs. This analysis not only reveals the spatial distribution patterns of MAHs, but also hints at their potential ability to promote community interaction and cultural exchange. The calculation formula is Chen et al. (17):


$$G=100\times \sqrt{\sum_{i=1}^{n}{\left(\frac{P_{i}}{Q}\right)}^{2}}$$

G is the geographical concentration index; $P_{i}$ represents the number of MAHs in the i th province; n is the number of provinces (cities, autonomous regions); Q is the total number of Chinese MAHs. The value of G is [0,100], the larger the value of G , the more concentrated the distribution of MAH. A smaller value of G indicates that the distribution tends to be dispersed.

### 2.4 Disequilibrium index (DI)

The imbalance index calculated by Lorentz curve method was used to analyze the distribution balance of MAHs in each region. This analysis can reveal whether the distribution of MAHs in the whole country is balanced, and then reflect the differences in the inheritance and development of MAC among different regions of China, which provides an important basis for reasonable planning and optimization of the layout of MAHs. The value of this index varies between 0 and 1, and its value reveals the degree of equilibrium or imbalance in the distribution of MAHs. The calculation formula is (18):


$$S=\frac{\sum_{i=1}^{n}Y_{i}-50(n+1)}{100\times n-50(n+1)}$$

n indicates the number of provinces, $Y_{i}$ is the accumulated percentage of the i th digit, The value of S is between 0 and 1, and the increase of the value indicates that the spatial distribution of MAHs is more unbalanced. When S =0, it means that MAHs are evenly distributed in each province, and when S =1, it means that MAHs are all clustered in a certain province.

### 2.5 Kernel density analysis (KDA)

Kernel density analysis was used to show the spatial density and distribution differences of MAHs. By revealing the density of MAHs in different regions, this method can help us understand the specific effects of geographical location, transportation convenience, and local population size on the distribution of MAHs. The calculation formula is (19):


$$f_{h}(x)=\frac{1}{\mathrm{nh}}\sum_{i=1}^{n}(\frac{x-x_{i}}{h})$$

$K(\frac{x-x_{i}}{h})$ is the kernel density function; $x-x_{i}$ is the distance from x to $x_{i}$ ; h represents the search radius and is greater than 0; The larger the value of $f_{h}(x)$ , the denser the distribution of MAHs.

### 2.6 Ordinary least squares (OLS)

To explore the formation mechanism behind the spatial production representation of contemporary MAHs, the main analysis tool used in this paper is Ordinary Least Squares, which is selected based on its extensive application and verification in classical regression analysis. This method was developed by Adrien-Marie Legendre and Carl Friedrich Gauss. The core of this method is to minimize the sum of squares of the error terms to estimate the unknown parameters in the linear regression model. The degree of influence of each independent variable on the dependent variable is visually displayed. Considering the relationship between the spatial production representation of Chinese MAHs and the multiple variables of the underlying formation mechanism explored in this study, OLS method will be more suitable for its accuracy and systematicity of ensemble linear estimation. Specifically, by constructing the number of MAHs as the dependent variable and the coherent external social environmental factors as the independent variable, OLS can effectively reveal how these variables jointly affect the spatial pattern of MAHs, to provide an important strategic reference for optimizing the inheritance and development space of MAC.

### 2.7 In-depth interviews (IDI)

From September 2024 to March 2025, we conducted semi-structured interviews with 10 martial arts halls across mainland China, employing purposive sampling to ensure comprehensive representation across five dimensions: geographic distribution (Eastern, Central, and Northern regions), urban hierarchy (first- to third-tier cities), operational scale (small traditional schools to large commercial chains). The interview protocol addressed five thematic modules: organizational management, spatial location decision-making, operational strategies, cultural transmission methods, and external environmental challenges. The interviews provided in-depth perspectives and rich details for understanding the micro-mechanisms underlying the spatial distribution of MAHs (see Table 2).

**Table 2:** Summary of interview information of martial arts halls.

Number	Name	Region	Interviewee
1	Elite MAH	Qingdao of Shandong Province	Cao**
2	New Star MAH	Jiaozuo of Henan Province	Wang**
3	Hongyang MAH	Changsha of Hunan Province	Liu**
4	Yongchun MAH	Shenzhen of Guangdong Province	Yuan*
5	Longyun MAH	Nantong of Jiangsu Province	Huang**
6	Weiguan MAH	Liaocheng of Shandong Province	Zhang*
7	Tongwei MAH	Weifang of Shandong Province	Liu**
8	Yongwan MAH	Anshan of Liaoning Province	Wang**
9	Longhu MAH	Shenyang of Liaoning Province	Sun*
10	Tiyuan MAH	Shenyang of Liaoning Province	Li**

The “*”, “**” is used to conceal the names of the respondents.

## 3 Result analyst

### 3.1 Spatial distribution patterns of MAHs

#### 3.1.1 The general distribution of MAHs was more in the east and less in the west

**Figure 1:**
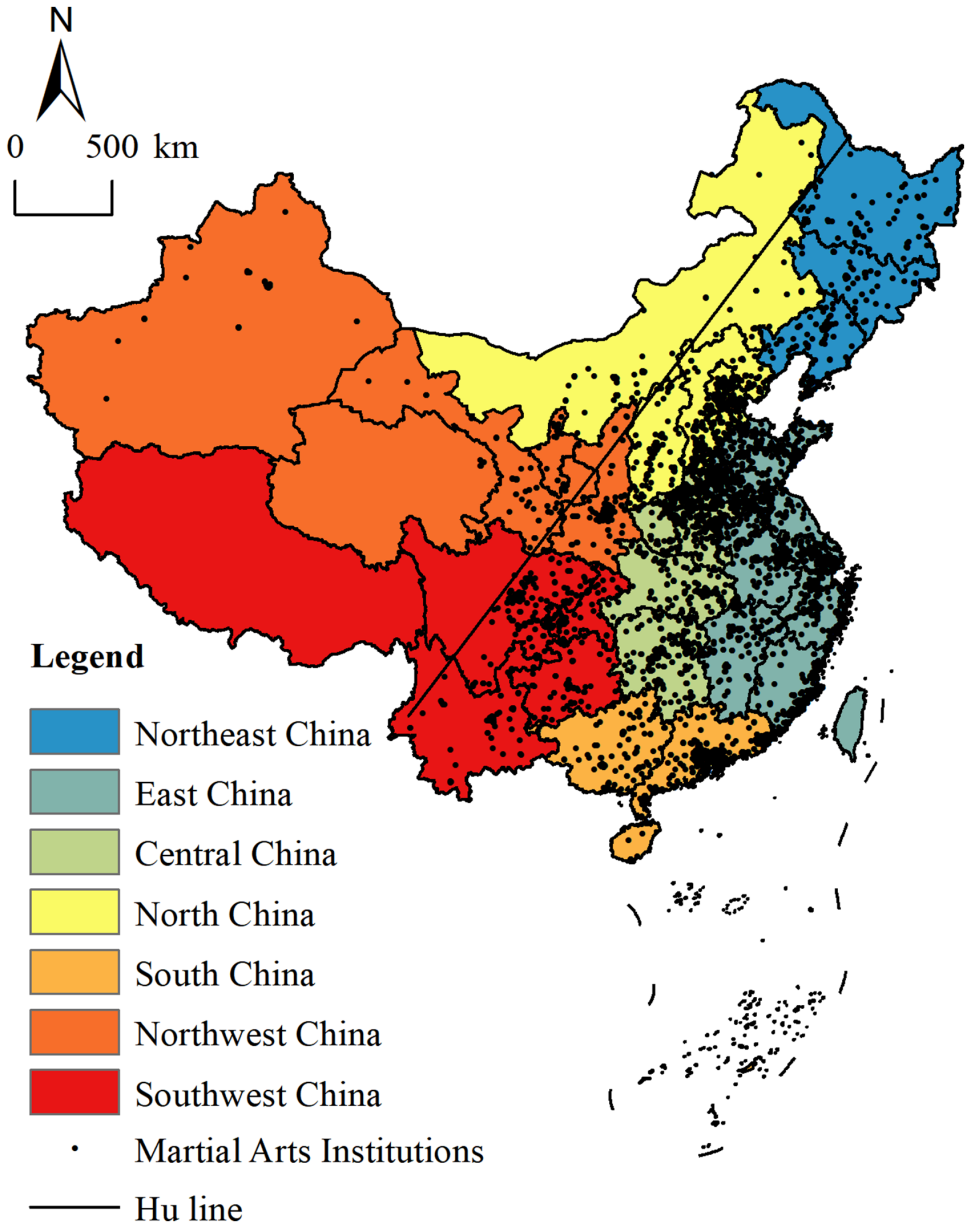
Spatial distribution of contemporary martial arts halls (http://bzdt.ch.mnr.gov.cn/browse.html?picId=%224o28b0625501ad13015501ad2bfc0288%22).

The spatial distribution of contemporary MAHs exhibits a pronounced east–west disparity, characterized by a marked concentration in the eastern region and relative sparsity in the west, thereby forming an agglomerative pattern of “more in the east, less in the west” (Figure 1). This distribution is not coincidental; rather, it is intrinsically linked to the broader geographical patterns of China’s population distribution. As delineated by the Hu Line (20) (China’s well-established population demarcation) the number and density of MAHs are significantly higher in the east than in the west. This mirrors the demographic settlement trend of “dense in the east, sparse in the west,” which has directly influenced and shaped the spatial configuration of MAHs across the country.

Through nearest neighbor index analysis, the pronounced spatial clustering of MAHs was revealed. The data indicated that the actual nearest neighbor distance was 7.53 km (compared to a theoretical distance of 28.58 km), yielding a nearest neighbor index of 0.263. Additionally, the Z-score was −102.4, which is statistically significant at the 5% level, thereby confirming the non-random, highly clustered spatial distribution of MAHs.

These findings demonstrate that the spatial arrangement of MAHs is not arbitrary but is shaped by a confluence of factors, including geographical conditions, socio-economic dynamics, and cultural influences. It is foreseeable that this spatial pattern will continue to evolve, potentially giving rise to even more pronounced agglomeration and centripetal tendencies, particularly along the west-to-east axis. The intensification of such spatial clustering suggests that the development trajectory of Chinese martial arts halls may exhibit increasing prosperity and diversification.

#### 3.1.2 Unbalanced density stratification among provinces of China

**Figure 2:**
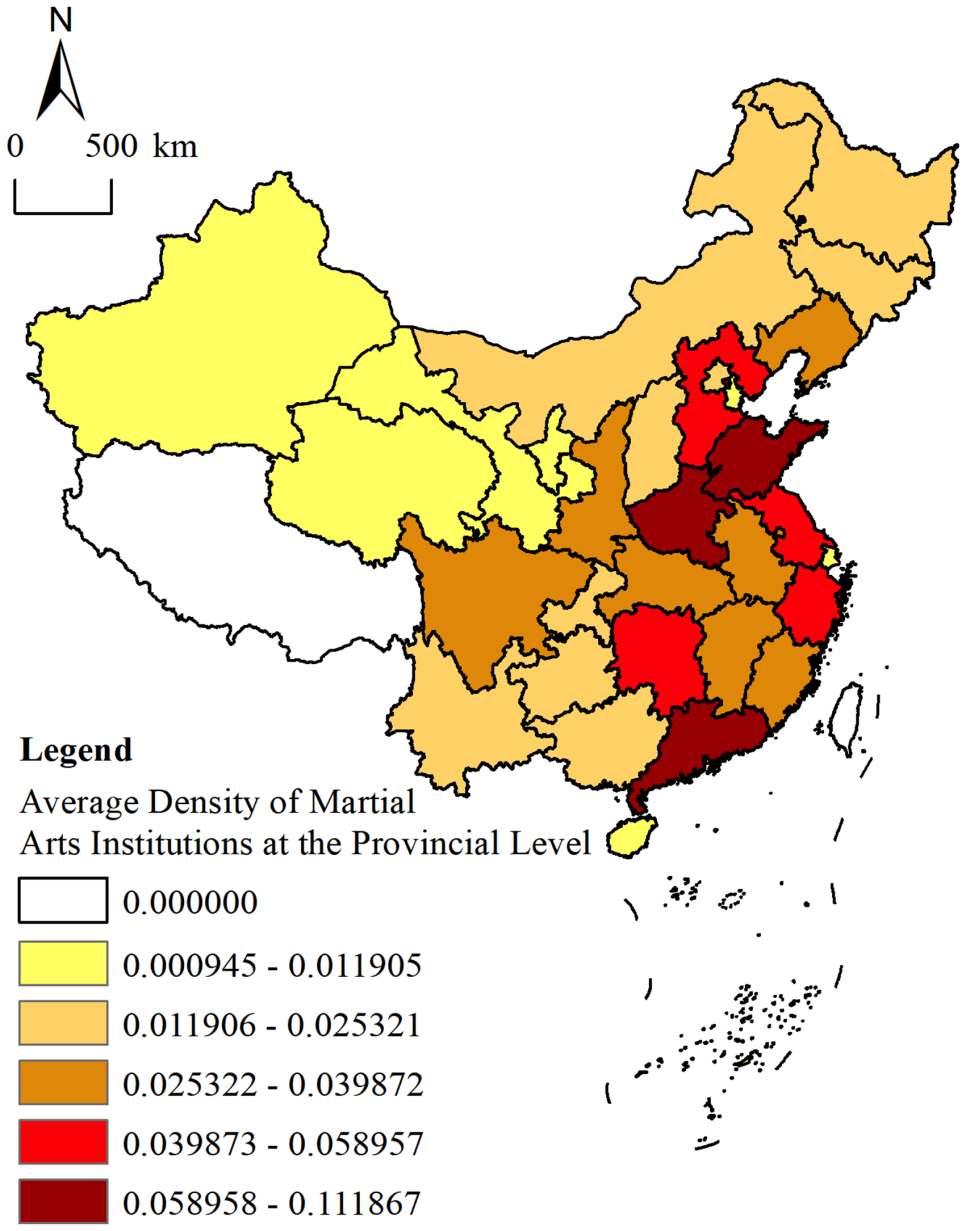
Spatial distribution density of contemporary martial arts halls in provinces (http://bzdt.ch.mnr.gov.cn/browse.html?picId=%224o28b0625501ad13015501ad2bfc0288%22).

Through a detailed analysis and computation of the geographical concentration index, the pronounced and uneven stratification of MAHs across Chinese provinces is revealed. The Jenks natural breaks classification method was employed to divide the provincial distribution of MAHs into five distinct levels (Figure 2). At the highest level, Shanghai ranks first in terms of average MAH count, followed by Beijing, Tianjin, Shandong, Henan, and Jiangsu at the second level. The third tier comprises Liaoning, Hebei, Anhui, Zhejiang, and Fujian, while the fourth tier includes Shanxi, Shaanxi, Ningxia, Hubei, Hunan, Chongqing, and Jiangxi. The fifth level encompasses Heilongjiang, Jilin, Inner Mongolia, Gansu, Xinjiang, Qinghai, Sichuan, Guangxi, Guizhou, Yunnan, and Hainan.

**Figure 3:**
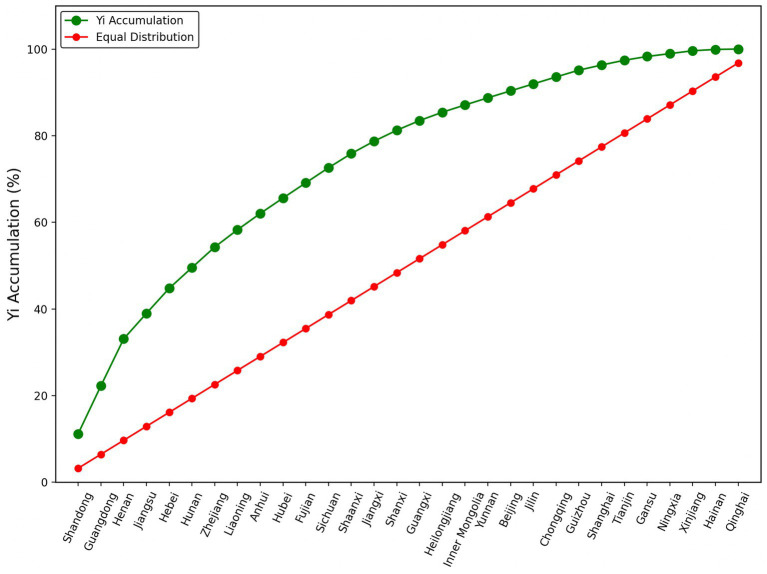
Lorentz curve of contemporary martial arts halls.

An in-depth examination of the data reveals that the calculated geographical concentration index ( G ) is 24.49, significantly exceeding the theoretical value of G0 18.25—an estimation based on the assumption of an even distribution of 170 MAHs across all provinces. This substantial deviation not only underscores the high degree of concentration at the provincial scale but also highlights distinct regional characteristics in the spatial distribution of MAHs. Further analysis reveals that the distribution imbalance index ( S ) is 0.57 (< 1) further confirming the spatially uneven distribution of MAHs. The upward convexity of the Lorenz curve is particularly pronounced (Figure 3), especially in provinces such as Shandong, Guangdong, Henan, and Jiangsu. In these areas, the number of MAHs accounts for over 40% of the national total, a statistic that not only underscores the regional disparities in distribution but also reflects the strong correlation between the presence of MAHs and the deep-rooted martial arts cultural traditions of these regions.

It is noteworthy that the spatial distribution characteristics of MAHs diverge significantly from those of conventional sports training institutions such as basketball, football, swimming, fencing, and physical fitness centers. Typically, the number of sports training facilities is positively correlated with the level of a city’s economic and social development. However, the distribution of MAHs exhibits a more intricate pattern. In addition to their higher density in economically developed regions, MAHs also demonstrate substantial clustering in areas with a deep-rooted martial arts tradition, such as Shandong and Henan. This distribution pattern reflects not only the regional cultural embeddedness of MAHs but also the strong interconnection between the preservation and development of martial arts—as a form of traditional cultural heritage—and the local cultural environment, along with prevailing socio-economic conditions. Therefore, the spatial configuration of MAHs is shaped not merely by economic indicators but also by long-standing regional cultural legacies and historical accumulation. In this context, provinces with high MAHs density should fully leverage the advantages of spatial clustering by promoting open and inclusive pedagogy, drawing upon the strengths of various boxings styles and MAHs, facilitating coordinated development across neighboring regions, and striving to establish regionally influential martial arts chain brands to prevent excessive convergence and homogenization (21). Conversely, provinces with lower MAHs density should focus on cultivating distinctive local martial arts styles while assuming a demonstrative and leading role within their respective regions. Simultaneously, efforts should be directed toward integrating MAHs with other sports resources to achieve resource complementarity and foster the synergistic development of related industries.

#### 3.1.3 Spatial distribution density

**Figure 4:**
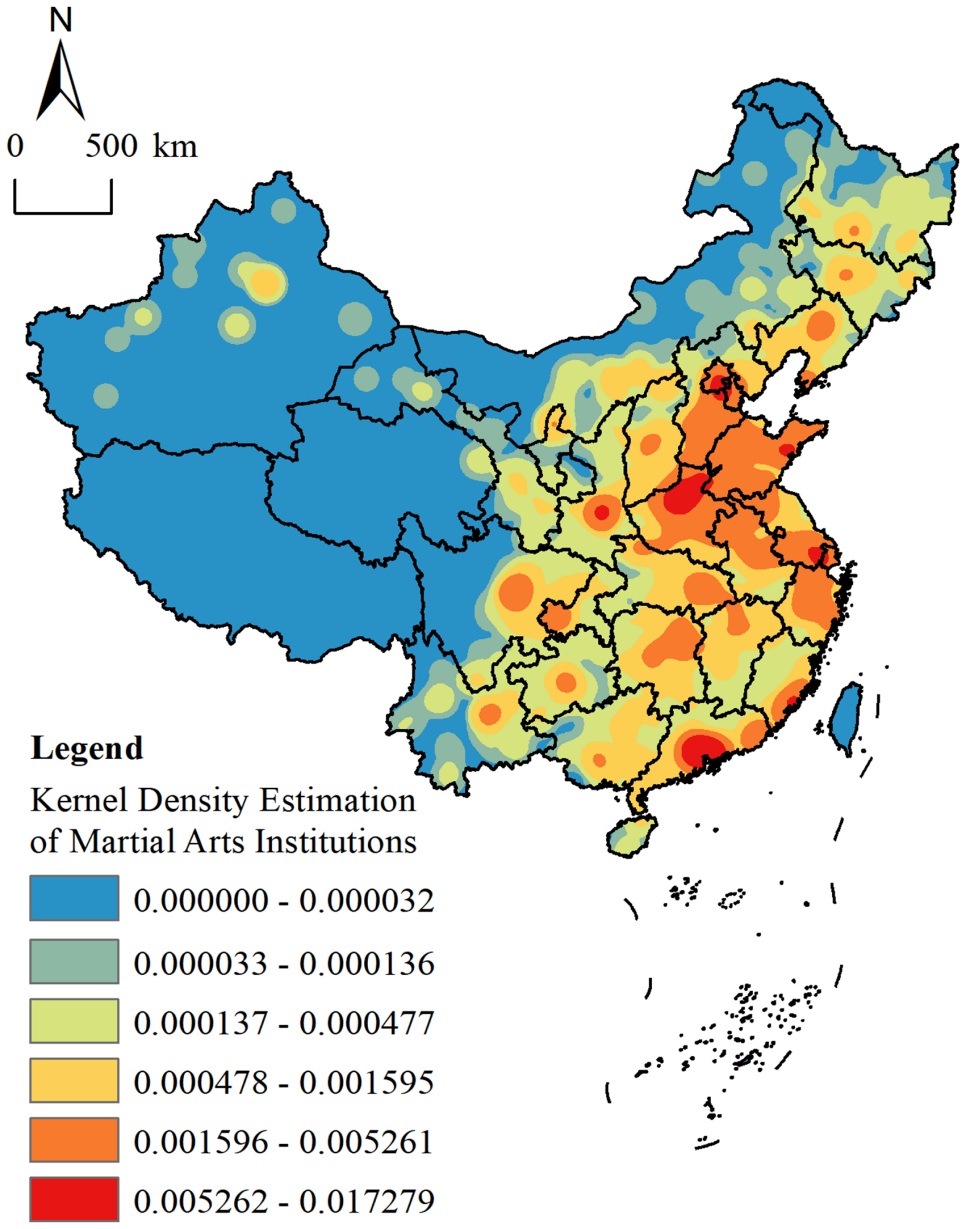
Kernel density of martial arts halls. (http://bzdt.ch.mnr.gov.cn/browse.html?picId=%224o28b0625501ad13015501ad2bfc0288%22).

Kernel density analysis revealed pronounced heterogeneity in the spatial distribution of MAHs across different regions (Figure 4). The density map illustrates a clear gradient of decline from the southeastern coastal areas toward the northwest, forming a concentrated and continuous spatial structure characterized by a “Four Core Ring-Core Group-Double Belt Area” model. The distribution of MAHs within the core areas is significantly influenced by factors such as regional economic development, market reach, consumer demand, and the accessibility of metropolitan transportation networks. In the strip-like contiguous zones, martial arts centers are predominantly located across the Loess Plateau, Yunnan-Guizhou Plateau, North China Plain, the middle and lower Yangtze Plain, and the Pearl River Basin-geographical regions forming part of the second geomorphological tier of China. Specifically, these belt-like contiguous areas include the junctions of Shanxi, Hebei, and Henan along both sides of the Taihang Mountains; the Qinghai-Gansu corridor surrounding the Wushan Mountains; and the extended belt encompassing the Xuefeng Mountains, Sichuan, Chongqing, and Guizhou. The formation of this spatial structure is shaped not only by geographical factors such as transportation accessibility and levels of economic development but also by the historical evolution and cultural imprint of martial arts traditions in various regions. Additionally, in the sub-core areas, provincial capitals such as Taiyuan (Shanxi), Chengdu (Sichuan), and others have emerged as relatively independent, single-core agglomerations, exhibiting a layered pattern of spatial distribution. This configuration aligns with the predictions of central place theory, suggesting that the spatial arrangement of MAHs is closely tied to the connectivity and hierarchical organization of the urban network.

It is evident that the spatial clustering of MAHs not only mirrors the geographical distribution and stylistic diversity of Chinese martial arts culture but also embodies the degree of social recognition and the cultural influence of martial arts as a regionalized cultural phenomenon (2). This is particularly prominent in the eastern coastal and central regions, where the dense concentration of MAHs reflects both the deep-rooted martial arts heritage and the high societal esteem for traditional Chinese martial arts. The spatial manifestation of this cultural phenomenon not only facilitates the transmission and innovation of martial arts traditions but also serves as a vital pillar supporting the enrichment of local socio-cultural diversity. Moreover, the distributional pattern of MAHs highlights the uneven dissemination of contemporary martial arts culture (10). High-density provinces such as Shandong, Guangdong, and Henan possess not only a rich martial arts heritage but also play a pivotal role in driving local economic development and cultural tourism. Conversely, the sparse presence of MAHs in western regions reflects, to some extent, the relative underdevelopment of these areas in terms of economic infrastructure and cultural resources, thereby constraining the broader dissemination and sustainable development of martial arts culture in these locales.

### 3.2 Determinants of MAHs distribution

#### 3.2.1 Index selection and data sources

The spatial production of contemporary martial arts halls (MAHs) is shaped by demographic, economic, cultural, and institutional forces. China’s settlement pattern—dense in the east and comparatively sparse in the west—imprints a “more-in-the-east, less-in-the-west” configuration on facility locations. Kernel-density and agglomeration analyses further reveal pronounced urban concentration, consistent with New Economic Geography arguments that urban environments amplify spillovers in demand, labor matching, and information flows (22). Guided by prior research on martial arts and the spatial distribution of organizational facilities (23–25). We extract six explanatory constructs: population scale, economic/consumption level, education level, sports-and-entertainment industry intensity, martial-arts policy support, and martial-arts cultural attention. Operational definitions appear in Table 3; the provincial number of MAHs is the dependent variable. An Ordinary Least Squares (OLS) regression model was employed to investigate the underlying mechanisms by which these factors influence the spatial distribution pattern of contemporary Chinese MAHs.

**Table 3:** Selection and meaning of influencing factors of spatial production representation of contemporary martial arts halls.

Explanatory variables	Measure index	Meaning	Expected impact
Population size	Resident population at the end of the year/10,000 People	Population density of the surrounding area of MAHs in each province. Shows the source market situation of MAHs	+
Economic level	Average annual consumption expenditure of urban households (Yuan/People)	Consumption level of people in each province Shows the economic situation of the MAHs area	+
Education level	College or above/10,000 People	Education level of province	+
Sports and entertainment industry	The number of Baidu Map POI in the category of sports and leisure services (excluding martial arts facilities)	The development degree and competitive intensity of alternative sports leisure and entertainment market in each province	+
Martial arts Policies	Number of martial arts related promulgations (items)	The level of support for martial arts by provincial and municipal governments. Shows the power of martial arts policy	+
Martial arts culture	Number of Baidu Index searches in recent five years (items)	The cultural atmosphere of martial arts in each province reflects people’s attention to martial arts	+

The data sources were as follows: (i) Socio-economics (population, education, consumption): “China Statistical Yearbook 2022” (26); (ii) Sports and entertainment industry: Python web crawler was employed to extract POI data for sports and leisure services from Baidu Maps (September 2024). The dataset included: (a) fitness centers and gyms; (b) swimming pools and aquatic centers; (c) ball sports venues (basketball, football, tennis, badminton); (d) dance and yoga studios; (e) other recreational sports facilities (skating rinks, climbing walls, bowling alleys). To avoid endogeneity, all martial arts-related facilities (identified through keywords: “wushu,” “martial arts,” “Kungfu,” “Taichi,” “boxing hall”) were systematically excluded from this variable. The final dataset comprised 47,832 non-martial arts sports and entertainment POIs across 31 provinces; (iii) Policy support: following Chen Sheng (27), full-text scraping of provincial/municipal government and Sports Bureau portals (2010–present) using martial-arts keywords. (iv) Cultural attention: Baidu Index^2^ Coordinates are standardized (WGS84); keyword sets (“wushu,” “martial arts,” “Kungfu,” “Taichi,” “boxing hall”) and cleaning rules are archived for reproducibility.

#### 3.2.2 Regression results

Model performance and diagnostics. Multicollinearity is modest (all VIFs < 6; mean VIF = 2.963). The model exhibits strong explanatory power (R^2^ = 0.915; adjusted R^2^ = 0.892) and is jointly significant at the 1% level (F-test, *p* < 0.01).

Initially, the selected variables were processed using SPSS, and the results of the multicollinearity test indicated that the variance inflation factor (VIF) values for all variables were below 6, with an average of 2.963. This suggests the absence of significant multicollinearity among the independent variables, thereby ensuring the reliability of the subsequent regression analysis. The regression model demonstrated a high degree of explanatory power, with an adjusted R2 value of 0.892, and it passed the F-test for overall significance at the 1% level. This indicates that the model accounts for approximately 89.2% of the variation in the spatial distribution of contemporary MAHs in China, offering strong empirical support for understanding the mechanisms underlying their spatial formation. Among the six explanatory variables, population size, education level, Wushu policy promotion, and Wushu cultural presence (Table 4) exerted a significant positive influence on the number of MAHs. In contrast, the prosperity of the sports and entertainment industry and the regional consumption level exhibited a negative correlation with MAH distribution. These findings underscore the complex interplay of socio-economic and cultural dynamics in shaping the spatial patterns of MAHs.

**Table 4:** OLS regression results of spatial production representation of martial arts halls.

Variable	Coefficient	T-value	*P*-value	VIF
Constants		3.420	0.002***	
Population size	0.847	6.124	0.000***	5.155
Economy level	−0.263	−3.062	0.006***	1.987
Culture, Sports and Entertainment	−0.220	−1.849	0.077*	3.801
Educational level	0.278	3.163	0.004***	2.087
Martial arts policy	0.142	1.628	0.067*	2.051
Martial arts culture	0.268	2.680	0.013**	2.697
R^2^	0.915			
Adjust R^2^	0.892			
*p* value	0.000***			

*, **, *** are at the significance level of 10, 5, 1%.

#### 3.2.3 Interpretation of key drivers

##### 3.2.3.1 Population density as dominant predictor

Population mass is the dominant predictor of MAH provision: the coefficient on population size (*β* = 0.847) indicates a steep elasticity of supply with respect to demand concentration. Densely settled provinces—Shandong, Guangdong, Henan—systematically host more MAHs, consistent with demand aggregation and agglomeration economies. Population operates dually as cultural substrate and addressable market; even modest participation rates applied to large demographic bases yield sizable client pools, underwriting venue viability and scale. Field evidence of deliberate micro-siting—co-location with schools and retail complexes—further reflects fine-grained targeting of pedestrian flows and neighborhood demographics. Heterogeneity in age structure segments demand and should drive program design: younger cohorts prefer dynamic, skill-intensive modalities (routines, weapons training), whereas older adults gravitate toward wellness-oriented disciplines (Taichi, Baduanjin). Accordingly, MAHs ought to offer differentiated, tiered curricula aligned with local demographic profiles. High density, however, also intensifies competition; providers in core markets must pursue product differentiation, service innovation, and precise marketing to sustain advantage. By contrast, in sparsely populated areas—where thin markets impede scale—strategic partnerships with community organizations and cultural/leisure enterprises can amplify visibility, extend reach, and enhance the societal salience of martial arts.

##### 3.2.3.2 Dual effects of economic development

Conditioning on other covariates, regional economic development is negatively associated with MAH density (*β* = −0.263), indicating that the economy–venue nexus is non-linear and context-contingent rather than monotonic. The effect operates through several intertwined channels that, at higher development levels, tend to crowd out traditional training venues even as purchasing power rises. (1) Cost—space pressures. In economically developed regions, commercial rents and ancillary operating costs escalate, compressing viable floor area or pushing halls to peripheral locations and thereby reducing accessibility and catchment quality. As one director in Anshan observed, “City-center rent is high, so our usable area keeps shrinking.” This spatial economics constraint forces MAHs to either relocate to peripheral areas or reduce their operational scale, both of which limit accessibility and market reach. (2) Educational time budgets and opportunity costs. Economic prosperity is accompanied by heightened academic competition, reordering household priorities toward exam preparation, and narrowing discretionary time for martial training—especially after upper primary grades. A Shenzhen operator noted, “Students focus on studying… they have little time.” This educational intensification crowds out time for martial arts training, particularly after grade four when academic pressure escalates. (3) Leisure diversification and substitution. Economic development reconfigures leisure ecologies on both the demand and supply sides of martial-arts training. Although rising incomes expand the budgetary capacity for cultural consumption, they simultaneously inflate the opportunity cost of time and proliferate low-commitment, high-entertainment alternatives. In amenity-rich, high-consumption regions, traditional MAHs therefore struggle to sustain salience, competing directly with video games, virtual-reality experiences, and experiential venues such as indoor surfing and skiing. On the supply side, the sector remains atomized and under-scaled: most MAHs operate at small sizes, lack systematic market intelligence, and display limited strategic agility. The regulatory compliance burden—tightened in the wake of the national “double-reduction” policy—further elevates fixed costs (licenses, deposits, fire-safety inspections, building/occupancy certificates), a pressure that stakeholders in developed jurisdictions repeatedly characterize as “particularly troublesome.” Against the backdrop of rapid socio-economic transformation and the shift from production to consumption-driven growth, this diversification yields a classic substitution dynamic: attention and discretionary time are reallocated away from skill- and time-intensive martial practice toward convenience-oriented leisure. Targeted interventions that lower spatial and regulatory frictions—e.g., rent abatements, school–MAH partnerships that internalize travel/time costs, off-peak program design, and streamlined compliance regimes for micro-providers—are likely prerequisites for translating economic prosperity into denser and more equitable MAH provision.

##### 3.2.3.3 Cultural salience and institutional density

Martial arts constitute a living form of intangible cultural heritage whose meanings are reproduced through practice, place, and pedagogy. Unsurprisingly, cultural salience and institutional presence are tightly coupled: our estimates show a positive association between the cultural-attention variable and MAH provision (*β* = 0.268), indicating that where martial arts command greater public attention, the organizational infrastructure that sustains them is denser. In other words, cultural vitality and facility distribution are mutually reinforcing rather than merely co-occurring phenomena. This linkage is spatially variegated. Provinces with deep martial traditions—Henan, Shandong, among others—exhibit a dense network of halls, underpinned by an accumulation of symbolic capital, training lineages, and event calendars. These ecosystems are not culturally uniform: Taijiquan in Henan exemplifies the philosophical precept of “using softness to overcome hardness,” aligning with yin–yang harmonics; Shaolin practice fuses corporeal discipline with Buddhist cultivation; Shandong styles, inflected by Confucian ethics, emphasize the unity of de (martial virtue) and technique; Fujian Southern Boxing, shaped by maritime exchange, privileges agility and fluid combinations. Such stylistic geographies embed local values and aesthetics into everyday training, thereby imprinting regional cultural logics onto the spatial layout of MAHs. Mechanistically, cultural salience operates through multiple, complementary channels. First, at the demand margin, higher public recognition—amplified by rising cultural confidence and health awareness—broadens the pool of potential participants and elevates parental willingness to invest in heritage-linked extracurriculars. Second, on the supply side, instructor reputation, credentialing, and lineage affiliations function as trust heuristics in markets characterized by information asymmetry, lowering search and switching costs for households and supporting organizational viability. Third, at the meso-institutional level, local associations, festivals, and heritage designations create “institutional thickness,” stabilizing calendars, consolidating resources, and anchoring MAHs within wider civic and educational partnerships. By contrast, regions where martial traditions are historically thinner—e.g., parts of Northwest China—often lack these reinforcing structures, and MAH presence remains comparatively sparse. Positioned at the intersection of tradition and modernity, MAHs thus serve as platforms of transmission and innovation: repositories of technique and philosophy, but also laboratories for the creative transformation of intangible heritage. Realizing this potential requires purposeful activation of endogenous cultural resources (lineage, repertoire, ritual), enhancement of public-service functions (school partnerships, community programming), and integration with contemporary communication channels. In sum, consolidating the cultural foundations that confer meaning and prestige is not ancillary to spatial planning; it is constitutive of a sustainable, place-sensitive ecology for the development and diffusion of Chinese martial arts.

##### 3.2.3.4 Policy as enabling but not deterministic

Policy planning functions as the architectonic layer of MAH spatial production: it sets the rules of the game, coordinates actors, and calibrates incentives. Empirically, however, its marginal effect is modest at the provincial aggregate (*β* = 0.142), suggesting that top-down frameworks enable but do not by themselves determine local provision. In short, policy operates primarily through the creation of meso-level conditions—standards, certification, event calendars, and financing channels—whose impact is mediated by implementation capacity and urban market frictions. At the national level, a coherent instrument mix has emerged. The Intangible Cultural Heritage Protection Law consolidates legal protection and earmarked funds. The “Wushu Industry Development Plan (2019–2025) (6)” articulates an integrated system linking education, performance, and commercialization; and the “14th Five-Year Sports Development Plan (28)” elevates the visibility and cultural influence of traditional sports. This top-down policy coherence supplies strategic direction and stable expectations for stakeholders across the heritage–sport–tourism nexus. At the subnational level, differentiated pathways illustrate the logic of place-sensitive governance. Guangdong leverages Lingnan lineages through heritage listing and tourism branding; Zhejiang combines capital investment with institutional platforms (e.g., martial-arts centers) to systematize local styles; Henan mobilizes its historical endowment via experience centers and themed parks. These initiatives increase institutional thickness—associations, venues, events—yet their effects on neighborhood-level accessibility vary with administrative competence, land and rental costs, and the depth of civic partnerships. Crucially, an implementation gap persists. Field evidence indicates low policy salience among operators, fragmented publicity, and escalating compliance burdens in the wake of “double reduction (5)”—licenses, deposits, fire-safety inspections, and occupancy certificates—that disproportionately tax small, community-based halls. In parallel, the exclusion of martial arts from high-stakes school sports tracks in some jurisdictions weakens parental incentives and depresses stable enrollment, thereby attenuating the very transmission pathways MAHs are designed to sustain. Although the “One School, One Specialty” initiative has raised visibility, interview data suggest its effects are blunted by policy–education incoherence at the local level and by uneven street-level administrative capacity. In summary, policy planning is necessary but not sufficient: its leverage turns on where and how instruments are delivered. A responsive, localized, and culturally attuned regime—one that reduces spatial and regulatory frictions (e.g., streamlined licensing, calibrated compliance, space-cost relief) while forging durable school–MAH linkages—offers the most credible route to a sustainable, place-sensitive MAH spatial ecology and to the modernization and internationalization of Chinese martial arts.

##### 3.2.3.5 Competitive substitution from sports and entertainment

Consistent with the baseline estimates (*β* = −0.220), the expansion of the sports–leisure complex is negatively associated with MAH density, reflecting a structural substitution of attention, time, and space rather than a simple short-run fluctuation. Three mechanisms are salient.

(1) Demand-side diversification and substitution. Rapid income growth has generated amenity-rich urban ecologies in which low-commitment, high-entertainment options—video games, VR experiences, indoor surfing/skiing—compete directly with time- and skill-intensive martial training. These formats better match preferences for immediacy, interactivity, and convenience, thereby reallocating discretionary time budgets away from traditional practice. Interview evidence further indicates intense competition from Taekwondo, football, and basketball, whose accessible rules and strong sociality accelerate adoption. (2) Product thresholds and participation frictions. The MAH model typically entails high entry requirements (flexibility, coordination, repetitive foundational work) and long horizons to visible progress, while the profusion of styles can induce choice overload among novices. Absent modular on-ramps and clear skill ladders, prospective learners face elevated psychological and opportunity costs relative to mainstream team sports. (3) Capital allocation and fixed costs. Most MAHs remain under-scaled and weakly branded, limiting their appeal to investors seeking rapid, platform-style growth. Capital scarcity constrains modernization (space, equipment, digital delivery), while compliance overheads—tightened post “double reduction” (licenses, deposits, fire safety, occupancy certification)—raise fixed costs that disproportionately burden small, community providers. To compete credibly in diversified leisure markets while preserving heritage value, MAHs should (a) re-engineer curricula into modular, competency-based pathways with micro-credentials to shorten time-to-benefit; (b) adopt hybrid O2O delivery (trial classes, short-form instructional media, live streams) to lower search and switching costs; (c) differentiate service portfolios (wellness/rehabilitation, after-school programs, festival/event formats, tourism tie-ins) to broaden demand; (d) pursue brand consolidation and instructor credentialing to reduce information asymmetry; and (e) leverage policy instruments—rent abatements, school–MAH shared-use agreements, and proportional, streamlined compliance—for cost relief and central-area access. In short, competition is multifaceted—behavioral, spatial, and financial. A strategy that marries product innovation and brand discipline with calibrated public supports can convert rivalry into complementarity, sustaining MAHs as living conduits of intangible cultural heritage within an increasingly diversified leisure economy.

##### 3.2.3.6 Education as cultural catalyst

The widespread advancement of education has not only reshaped the intellectual landscape of contemporary society but also played a pivotal role in revitalizing traditional culture—particularly the practice of martial arts. A statistical analysis reveals a positive correlation (*β* = 0.278) between educational attainment and public recognition of martial arts, suggesting that higher levels of education are associated with greater appreciation of this cultural heritage. This relationship can be examined from several perspectives. First, education fosters a renewed appreciation of the nation’s rich cultural heritage, including martial arts, which embodies a unique synthesis of historical, philosophical, and physical elements. As educational standards rise, individuals develop a deeper understanding of the multifaceted value of martial arts—not merely as a form of physical training, but also as a cultural medium for heritage preservation, personal discipline, and self-defense. In a society where tradition and modernity continually intersect, educational advancement has spurred a renewed quest for cultural roots. Consequently, martial arts is increasingly perceived not only as a means of fitness but also as a conduit for cultivating national identity, pride, and a sense of belonging. This shift has given rise to a trend wherein highly educated individuals increasingly invest time and resources in martial arts practice, motivated by a pursuit of physical well-being and spiritual fulfillment. Field investigations further show that students who engage in martial arts training often come from well-educated families. Parental education levels significantly influence children’s motivation to participate in martial arts, since well-educated parents are more likely to value traditional culture and encourage their children to engage in these cultural practices. Moreover, as the overall quality of education continues to rise nationwide, an increasing number of families recognize the role of martial arts in fostering both physical fitness and moral development. Second, advancements in education have accelerated the modernization and professionalization of martial arts halls (MAHs). In regions with abundant educational resources—such as Jiangsu and Shanghai—the overall competency of martial arts instructors has markedly improved. These professionals are increasingly adept at integrating modern management practices to align their programs with market demands. For example, interviews with MAH operators reveal that those with higher education levels are more proactive in leveraging digital platforms (e.g., WeChat official accounts, TikTok, and similar social media) to broaden their reach and influence. They readily adopt strategies such as live-streamed classes, short-form instructional videos, and interactive online engagement to enhance visibility and attract new participants. Third, educational authorities have launched a “Martial Arts into Schools” initiative that has significantly boosted young people’s enthusiasm for martial arts. Coupled with newly implemented academic burden-reduction policies, a growing number of schools are now partnering with MAHs to provide students access to martial arts training. This model not only alleviates the shortage of physical education resources in schools but also creates new opportunities for martial arts programs to develop and integrate into the formal education system. In this context, education serves as a form of soft power that revitalizes martial arts by strengthening both individual and collective identification with traditional martial culture. Looking ahead, the ongoing integration of educational and cultural systems is expected to establish martial arts as a vital bridge between the national spirit and contemporary life, thereby enabling its wider dissemination and sustainable development throughout society.

## 4 Discussion

### 4.1 Spatial patterns of MAHs in the context of sports geography

Our findings demonstrate that MAHs exhibit pronounced east–west clustering and urban concentration, echoing classical principles of sports geography. Bale’s pioneering framework on sports landscapes emphasizes that sporting facilities are not randomly distributed but are shaped by demographic density, economic development, and cultural infrastructure (12). The spatial pattern of Chinese MAHs—dense in eastern coastal provinces (Shandong, Guangdong, Henan) and sparse in the western interior—aligns with Bale’s observation that sports facilities cluster where market demand, institutional support, and cultural capital converge. However, our study extends this framework by revealing the dual role of economic development: while wealth enables facility provision, it also intensifies competition from alternative leisure options and elevates operational costs, thereby creating crowding-out effects. This paradox is less prominent in Bale’s Western-centric analysis but emerges as critical in contexts where traditional cultural practices compete with globalized entertainment industries. Recent comparative studies of sports venues distribution in China further contextualize our findings. Zhang et al.’s analysis of sports venues in urban China highlights similar agglomeration dynamics, wherein facility density correlates with urbanization rates and disposable income. Yet MAHs diverge from commercial fitness centers in their cultural embeddedness: they function not merely as service providers but as repositories of intangible heritage. This dual identity—commercial yet cultural—subjects MAHs to institutional pressures (heritage preservation mandates) and market pressures (profitability imperatives) simultaneously. Our regression results confirm that cultural salience (β = 0.268) significantly predicts MAH density, suggesting that heritage value operates as a distinct determinant alongside conventional socioeconomic variables. This finding contributes to sports geography by demonstrating that culturally embedded facilities require analytical frameworks that integrate both market logic and heritage politics.

### 4.2 MAHs as sites of intangible cultural heritage transmission

UNESCO’s framework for safeguarding intangible cultural heritage emphasizes the importance of ‘living transmission’—the ongoing practice and pedagogy that keep traditions vibrant rather than museumified. MAHs exemplify this principle: they are not static archives but dynamic training venues where techniques, philosophies, and lineages are enacted daily. Our fieldwork confirms that MAHs serve multiple heritage functions: (1) skill transmission through master-apprentice pedagogies, (2) community anchoring via local festivals and competitions, and (3) cultural innovation through curriculum adaptation (e.g., integrating wellness-oriented Taichi for older adults). However, our findings also reveal tensions within the heritage safeguarding paradigm. The negative correlation between regional economic development and MAH density (*β* = −0.263) suggests that market-driven urbanization can paradoxically undermine heritage transmission. High real estate costs in developed cities force MAHs to peripheral locations, reducing accessibility for urban populations who constitute the primary clientele for heritage engagement. This spatial marginalization contradicts UNESCO’s emphasis on community-based safeguarding, wherein heritage practices should be embedded within everyday life rather than relegated to tourist-oriented or niche spaces. Comparative research on intangible heritage transmission offers instructive parallels. Zhang et al.’s study of traditional Chinese opera troupes documents similar challenges: rising costs, declining public interest among youth, and competition from digital entertainment. Wang argues that effective heritage safeguarding requires hybrid strategies—combining state subsidies (to buffer market pressures), school partnerships (to cultivate youth engagement), and digital platforms (to expand reach) (29). Our policy recommendations align with this multi-pronged approach, advocating for rent abatements, school–MAH collaborations, and online-offline integrated delivery models. Such interventions address the structural contradictions between heritage preservation and market viability, ensuring that MAHs remain accessible and relevant.

### 4.3 Contributions to new economic geography and spatial economics

From a theoretical standpoint, our findings contribute to New Economic Geography (NEG) by demonstrating how cultural factors interact with conventional agglomeration forces. Yang and Hamaguchi’s framework posits that agglomeration arises from increasing returns to scale, labor market pooling, and knowledge spillovers (18). While these mechanisms apply to MAHs—urban areas offer larger client bases, instructor networks, and event ecosystems—our study reveals an additional layer: cultural salience as an agglomeration attractor. Provinces with deep martial traditions (Henan, Shandong) sustain dense MAH networks not solely because of economic scale but because cultural capital lowers information asymmetries (instructor reputation as trust signal), stabilizes demand (heritage tourism, intergenerational participation), and attracts public investment (heritage designation funds). This cultural dimension introduces path dependence into facility location. Unlike generic commercial services that can relocate to optimize market access, MAHs are spatially ‘sticky’ due to lineage ties, historical sites, and symbolic landscapes (e.g., Shaolin Temple in Henan). This stickiness implies that spatial inequality in MAH provision may persist even as economic development diffuses westward, unless deliberate policies cultivate cultural infrastructure in under-served regions. Our findings also speak to spatial economics literature on competition and substitution. The negative effect of the sports–entertainment industry on MAH density (*β* = −0.295) reflects a zero-sum competition for discretionary time—a finite resource in urbanized, high-income contexts. This substitution dynamic differs from complementary agglomeration (e.g., restaurants clustering near theaters to capture foot traffic). Instead, MAHs and modern leisure venues compete for the same demographic (youth, urban professionals) within constrained time budgets, leading to spatial displacement. Policy interventions that reduce MAH operational frictions (streamlined licensing, shared-use school facilities) can shift this competition from zero-sum to coexistence, enabling MAHs to carve differentiated niches (cultural authenticity, wellness focus) within diversified leisure ecologies.

### 4.4 Limitations and future research directions

Several limitations warrant acknowledgment. First, our Baidu Index measure of cultural attention, while validated in prior health and tourism research, captures search behavior rather than actual participation. Future studies should triangulate this with alternative indicators—practitioner registration data, cultural event attendance, martial arts association membership—to more precisely gauge cultural engagement. Additionally, regional differences in internet penetration may introduce measurement bias, particularly in rural and western provinces where digital access lags.

Second, our analysis aggregates MAHs at the provincial level, masking intra-provincial heterogeneity. Urban–rural disparities within provinces (e.g., concentrated MAHs in Zhengzhou vs. sparse presence in Henan’s rural counties) remain unexplored. Fine-grained, city-level or county-level analyses could reveal localized drivers and inform spatially targeted policies. Finally, our study focuses on supply-side determinants (population, policy, cultural capital) but does not systematically examine demand-side preferences. Household survey data on martial arts participation motivations, willingness-to-pay, and perceived barriers would complement our facility-level analysis and inform product innovation strategies. Future research should also explore comparative international dimensions. How do MAHs’ spatial patterns and survival strategies compare to traditional martial arts facilities in Japan (karate dojos), Korea (taekwondo dojangs), or Brazil (capoeira schools)? Cross-national comparisons could identify universally applicable heritage safeguarding mechanisms versus culturally specific adaptations, advancing global discourse on intangible cultural heritage transmission in market economies.

## 5 Conclusion and policy recommendation

### 5.1 Conclusion

This study examined the spatial distribution patterns and determinants of contemporary martial arts halls (MAHs) in China, integrating quantitative spatial analysis, regression modeling, and qualitative fieldwork. The analysis reveals pronounced regional disparities, complex socioeconomic drivers, and tensions between cultural heritage preservation and market pressures. Three main findings emerge: First, MAHs exhibit significant spatial agglomeration, with eastern provinces—notably Henan, Shandong, and Guangdong—forming major clusters, while western provinces remain sparse. Kernel density analysis identifies a “Four Core–Ring Core Group–Double Belt Area” structure, characterized by high-density cores (Henan, Shandong), radiating clusters (Jiangsu, Zhejiang, Guangdong), and north–south connectivity belts along urbanized corridors. This distribution mirrors China’s broader population and economic geography, confirming that MAH provision is tightly coupled with demographic concentration and urban development. Second, the spatial layout of MAHs is shaped by complex, multi-dimensional drivers. Population size emerges as the dominant predictor (*β* = 0.847), reflecting demand aggregation and agglomeration economies. Education level (*β* = 0.278) and cultural salience (β = 0.268) exert positive effects, underscoring the role of human capital and heritage valuation in sustaining traditional practice. Policy support, while enabling, shows modest direct effects (β = 0.142), suggesting that top-down frameworks require complementary local implementation to translate into facility provision. Conversely, regional economic development (β = −0.263) and sports–entertainment industry density (β = −0.295) exhibit negative associations, revealing crowding-out mechanisms: high real estate costs, intensified leisure competition, and elevated opportunity costs constrain MAH viability in affluent, amenity-rich contexts. Third, MAHs occupy a dual identity—as commercial service providers and as repositories of intangible cultural heritage—subjecting them to simultaneous market and institutional pressures. Field evidence confirms operational challenges: escalating compliance costs post-“double reduction” policy, spatial displacement to peripheral locations due to rent pressures, and substitution effects from modern entertainment alternatives. Yet MAHs also demonstrate adaptive capacity, leveraging digital platforms (online instruction, social media marketing) and hybrid models (school partnerships, wellness-oriented programming) to expand reach and diversify revenue. These findings highlight the need for policy frameworks that recognize MAHs’ cultural value while supporting their market sustainability.

### 5.2 Policy recommendation

Against the backdrop of evident regional disparities, policy intervention must shift from uniform administrative promotion to differentiated, region-specific governance. To optimize spatial equity and cultural sustainability, we propose a multi-tiered strategy: In eastern, demographically concentrated cores, prioritize curricular modernization, diversified instructional content, and partnerships with schools and cultural organizations to meet pluralistic leisure-culture demand. Support brand consolidation, instructor credentialing, and hybrid O2O delivery models to enhance competitiveness in saturated markets. In western and under-served regions, emphasize safeguarding of intangible martial heritage, support for community-based halls, and targeted fiscal and institutional instruments (e.g., rent abatements, instructor training funds, streamlined licensing). Leverage heritage designation and tourism integration to create sustainable revenue streams that buffer market thinness. At the national level, establish inter-provincial resource-sharing mechanisms (credential portability, competition circuits, mobile training camps) and inclusive public-service platforms to raise baseline accessibility. Accelerate provision in sub-high-density cores and township-level nodes to improve coverage and catalyze leisure-sport and sports-tourism synergies. Finally, calibrate regulatory compliance to proportionately burden large commercial chains while easing fixed costs for micro-scale, community halls, ensuring that heritage safeguarding does not inadvertently exclude grassroots providers.

## Data Availability

The original contributions presented in the study are included in the article/Supplementary material, further inquiries can be directed to the corresponding author.

## Footnotes

^1^ https://api.map.baidu.com/lbsapi/getpoint/

^2^ http://index.baidu.com
